# Impairments of Visuospatial Attention in Children with Unilateral Spastic Cerebral Palsy

**DOI:** 10.1155/2018/1435808

**Published:** 2018-12-17

**Authors:** Gaétan Ickx, Samar M. Hatem, Inmaculada Riquelme, Kathleen M. Friel, Camille Henne, Rodrigo Araneda, Andrew M. Gordon, Yannick Bleyenheuft

**Affiliations:** ^1^Institute of Neuroscience, Université catholique de Louvain Brussels, Belgium; ^2^Faculty of Medicine and Pharmacy, Faculty of Physical Education and Physiotherapy, Vrije Universiteit Brussel, Brussels, Belgium; ^3^Brugmann University Hospital, Brussels, Belgium; ^4^Research Institute on Health Sciences (IUNICS-IDISBA), University of the Balearic Islands, Palma de Mallorca, Spain; ^5^Department of Nursing and Physiotherapy, University of the Balearic Islands, Palma de Mallorca, Spain; ^6^Burke Neurological Institute, White Plains, NY, USA; ^7^Weill Cornell Medicine, New York, NY, USA; ^8^Teachers College, Columbia University, New York, NY, USA

## Abstract

**Aim:**

This observational study aimed at assessing the prevalence of visuospatial attention deficits in children with unilateral spastic cerebral palsy (USCP), taking into consideration the affected hemibody and the localization of the brain lesion.

**Method:**

Seventy-five children with USCP were assessed with four visuospatial attention tests: star cancellation, Ogden figure copy, line bisection, and proprioceptive pointing.

**Results:**

A majority (64%) of children with USCP presented a deficit in at least one test compared to the reference values. The alterations observed in children with left or right USCP were related to egocentric or allocentric neglect, respectively. Children with cortico/subcortical lesion presented more often visuospatial attention deficits than children with periventricular lesion. Visuospatial attention deficits were not associated with brain lesion locations.

**Interpretation:**

Visuospatial attention deficits are prevalent in children with USCP and should be taken into account during their rehabilitation process. The present results shed new light on the interpretation of motor impairments in children with USCP as they may be influenced by the frequent presence of visuospatial deficits.

## 1. Introduction

Cerebral palsy (CP) results from brain lesions occurring during prenatal, perinatal, or early postnatal life. Cerebral palsy's overall prevalence is 2 per thousand live births and highest in children born before 28 weeks of gestation [1]. One of the most common subtypes of CP is unilateral spastic cerebral palsy (USCP) which represents up to 34% of all cases [1–5]. The main consequence of USCP is motor impairment which depends on the timing, size, and localization of the lesion as well as on the child's cerebral reorganization and recovery [6]. Additional impairments include deficits in sensory and cognitive function as well as sensory-motor integration [7, 8]. Visuospatial attention is the capacity of someone to attend to and to process stimuli in his surrounding space [9]. Visuospatial attention deficits are likely to be present in children with USCP, probably at least in part, influenced by the impact of the motor deficit over the attentional system [10], though they scarcely have been studied.

Visuospatial attention deficits have been widely studied in adult patients with acquired brain lesions and are mainly observed in lesions of the right hemisphere. They lead to hemineglect of the contralesional body and hemispace in 10 to 33% of patients [11–13]. Neuroimaging studies have shown a relationship between hemineglect and lesions located in the right temporoparietal junction (TPJ), as well as in certain areas of the frontal, parietal, and temporal lobe [14, 15]. In visuospatial attention, different frames of reference—either egocentric or allocentric—can be distinguished. Egocentric neglect is described with regard to the body midline of the patient (i.e., the patient neglects stimuli presented on one side of the hemispace referred to his own body midline) and allocentric neglect is described with regard to the midline of an object in the peripersonal or extrapersonal space (i.e., the patient neglects stimuli on one side of the object's midline). The egocentric visuospatial representation is important for movement planning and motor control during direct interaction between body and objects, while the allocentric representation is important for determining spatial references in the environment. The interaction between the allocentric and egocentric visuospatial representations allows for spatial processing [16, 17]. Both allocentric and egocentric visuospatial representations show a progressive maturation with age in typically developing children, with only the egocentric visuospatial representation reaching maturity upon adolescence [14, 18]. After stroke in adults, dissociations may appear between egocentric and allocentric neglect [14]. These dissociations may differ in function of the physical distance between the subject and the visuospatial attention test [19]. Despite the relevance of both ego-and allocentric spatial representations, studies in children with USCP have interpreted visuospatial assessments mostly with regard to the egocentric reference frame (cancellation tasks, figure copy, drawing, and exploration tasks). To our knowledge, no studies specifically have assessed allocentric visuospatial attention in a large sample of children with USCP.

Few studies have reported lateralized visuospatial attention deficits in children with early brain lesions. Trauner [20], in a study with a large sample of children with early brain lesions (*n* = 60) and typically developing (*n* = 36) children, reported evidence of spatial neglect in two-thirds of children with both left and right brain lesions. In this study, a board with toys was presented to toddlers and the localization of toys touched by the child was recorded. Other studies [21–23] also reported the presence of spatial neglect in children with a right or left early brain lesion using, for example, the teddy bear cancellation test. Another study focused on children with early left brain damage [24] and reported the presence of a correlation between the reorganization of language function in the right hemisphere and visuospatial performance in the star cancellation test. Yousefian et al. [25] observed contralateral neglect in children with perinatal stroke using the clock drawing test in comparison with a control group. Differences were reported according to the side of lesion and the age of children: younger children (6–8 years) with right hemispheric lesions had error patterns similar to adult patients with right hemispheric lesions [25]. Visuospatial attention appears as a dynamic process maturing with age in children with CP as well as in typically developing children [18, 25]. While contralateral neglect in adults is mainly observed with right hemispheric lesions, it may occur in children with right or with left hemispheric lesions. This suggests differences in the anatomical distribution and brain reorganization of visuospatial abilities between the developing and mature brain.

Independently from contralateral neglect, children with CP also have been reported to present deficits of executive functions and more specifically of global attentional control. One study showed attentional deficits in children with CP, as well as a lower performance of inhibition, working memory, and general executive function [26]. Another study reported the presence of global executive deficits in children with CP based on the Rey-Osterrieth complex figure and subtests of different executive functioning assessments [27]. Attentional deficits were reported as nonlateralized, though some differences were observed between children with left and right CP in inhibition/switching tasks.

Furthermore, visuospatial attention has been shown to be acquired in function of locomotor experience. Previous research has shown in toddlers that visuospatial attention improves with the development of walking abilities [28]. In children with CP, brain plasticity and reorganization are correlated with several functions such as motor abilities, language, and vision [29–33].

The aim of the present study is to investigate the prevalence of visuospatial attention deficits in a large sample of children with USCP, using both ego- and allocentric tests with regard to the affected hemibody. We hypothesized that many children with USCP would show abnormal values in both ego- and allocentric visuospatial attention tests. Detecting the presence of these deficits appears as important to tailor the rehabilitation process to each child and thus to improve his/her ability in everyday motor activities.

## 2. Method

### 2.1. Participants

Children with USCP (*n* = 75) were recruited by the MSL-IN Lab (Institute of Neuroscience, Université Catholique de Louvain, Brussels, Belgium) and the Center for Cerebral Palsy Research (Teachers College, Columbia University, United States) during four consecutive years (2013–2016). All children were participants of an intensive rehabilitation program and were assessed for the present research before starting the intensive rehabilitation programs.

The inclusion criteria were as follows: (1) aged between 5 and 18 years, (2) ability to grasp light objects and lift the more affected arm 15 cm above a table surface, (3) ability to follow instructions and complete testing, (4) attending school in the same grade as their typically developing peers of the same age, (5) Manual Ability Classification System [34] levels I, II, or III, and (6) Global Motor Function Classification System levels I, II, or III [35]. Exclusion criteria were as follows: (1) uncontrolled seizures, (2) orthopedic surgery or botulinum toxin injections less than twelve months before or within the study period, and (3) possibility of treatment/testing interference because of uncorrected visual problems (as described by their physician). No formal cognitive assessment was performed or used as inclusion criterion in the present study. Participants and caregivers provided informed consent. The study was approved by the Institutional Review Boards of the Teachers College, Columbia University, and of the Université Catholique de Louvain.

Brain MRI and ophthalmological assessments were not performed as part of this study. Given that the children were participants of an intensive rehabilitation program off-site from the hospital, there was no simple access to the technologies needed for brain imagery and ophthalmological evaluations within the time frame of the present study. Therefore, when previous MRI was available, brain lesions were classified by a neuroradiologist using the criteria of Krägeloh-Mann and Horber [36], allowing to define the origin/timing of their brain lesion (cortical malformation, periventricular lesion, or cortical/subcortical lesion). In addition, the localization of the lesion in the brain was described. In a subsample of the study population and not performed as part of this research, a previous detailed ophthalmological examination by an ophthalmologist (Cliniques Universitaires Saint-Luc, Université Catholique de Louvain, Brussels, Belgium) was available and retrieved from the medical file. This examination included assessment of visual function (visual acuity, visual field defect with Goldmann visual field perimetry, color perception, and refractive error), binocular vision (binocular vision with Worth's test and Bi-prism test, near point of convergence, eye motility assessment with Broad H, stereopsis and stereoacuity with TNO test, and strabismus with cover test), and finally ophthalmological health (examination of anterior and posterior segment). More specifically, in the Goldmann visual field perimetry, children have to maintain fixation on a central point; the fixation is controlled by a trained perimetrist, while a visual stimulus is moved around the patients' visual field. The children have to report by pressing a button, whether they can see the target or not. The visual field of the child is then plotted [37]. In the broad H test, the children are asked to follow a target (a penlight) which is moved in an H pattern to the edge of the binocular field. The ophthalmologist has to record any misalignments of the patients' eyes which could indicate eye motility deficits [38].

### 2.2. Assessment Tools

Four assessments of visuospatial attention were used: star cancellation, Ogden figure copy, line bisection, and proprioceptive pointing. The four visuospatial tasks were chosen for the following reasons: (1) reference values are available in same-aged typically developing children [18]; (2) the same tasks can be used in adults, ensuring the possibility of a follow-up in the transition from childhood to adulthood; (3) the tasks can be performed single-handedly with the less affected hand, limiting a bias due to sensorimotor deficit; (4) the tasks do not require any other material than paper and pencil; and (5) the tasks are language-independent. The latter two reasons were important in this study as children were included both in Belgium (French) and the US (English). Results of the visuospatial attention assessments were considered as abnormal when lying outside the range of normal values previously described for each age category [26].

#### 2.2.1. Star Cancellation

The test consists of an A4 sheet of paper with stars of two different sizes as well as distractor words which are semirandomly distributed. The child is asked to cancel all small stars. The following variables are recorded: the number of stars omitted on each side (left, right) and the total number of omitted stars [39]. The absolute difference between the number of left omitted stars and right omitted stars also is computed. The variable used to determine if a child with USCP presents with an abnormal value compared to reference values is the total number of omitted stars. Star cancellation mainly assesses egocentric neglect [19].

#### 2.2.2. Ogden Figure Copy

This test consists of a drawing copy task. The child is asked to copy a figure (a house and 4 trees). The score ranges from 0 (no omissions) to 4 (multiple omissions) [40] and is the variable used to determine if a child with USCP presents with an abnormal value compared to reference values. Ogden figure copy assesses both ego-and allocentric neglect [41].

#### 2.2.3. Line Bisection

The line bisection test consists of 2 pages with 10 lines of different lengths on each page. The child is asked to indicate the middle of each line by making a mark with a pencil. The deviation from the center, in percentage of half the line length, is computed with the following formula: deviation = (b − a)/a^∗^100, where *a* is half length of the line and *b* is the distance between the beginning of the line and the mark made by the child [42]. The variable used to determine if a child with USCP presents with an abnormal value compared to reference values is the average deviation (in percentage) from the center of each line. Line bisection test assesses allocentric neglect. An error towards the paretic side of space is recorded as a negative value.

#### 2.2.4. Proprioceptive Pointing

The child is blindfolded and seated in front of a table. A paper sheet with angled graduation lines (deviation in degrees) from a central point is aligned with the body midline of the child. The child is asked to point straight ahead on the table by moving his finger [43]. The pointing is performed three times. The variable recorded is the average deviation (mean of the three pointings in degrees) with regard to the child's body midline. This variable is used to determine if a child with USCP presents with an abnormal value compared to reference values. Proprioceptive pointing assesses egocentric neglect. A deviation towards the paretic side of space is recorded as a negative value.

## 3. Statistical Analysis

The descriptive statistic is as follows: a child with USCP was considered to have an abnormal value for any of the visuospatial attention tests if his/her result was outside the age-corrected reference values published previously [18].

Chi-square tests were used to investigate the association between demographic characteristics and the presence of abnormal visuospatial attention assessments, as well as to investigate the association between different abnormal visuospatial attention assessments. Chi-square tests were used to investigate the association between the presence of abnormal visuospatial attention assessments in different age groups (13 age groups from 5 to 17 yrs).

Chi-square tests were used to investigate the association between the presence of abnormal visuospatial assessments in children with left USCP vs. children with right USCP, in children taking antiepileptic drugs vs. children not taking antiepileptics, and in children with predominant periventricular brain lesions vs. children with cortico/subcortical brain lesions as well as between the different localizations of lesions.

Post hoc Bonferroni was used to correct for the multiplicity of tests.

Student *t*-test was used to compare intrasubject differences between omissions on one side and the other side of hemispace for the star cancellation test.

The statistical analysis package SPSS was used for all analyses. Significance level was set at *p* ≤ 0.050.

## 4. Results

### 4.1. Participants

The demographic and clinical data of the study sample are summarized in Table 1. The sample consisted of 75 children with USCP from 5 to 17 years old (mean = 9 y 3 m, SD = 2 y 11 m, 42 boys and 33 girls): 45 children with right USCP and 30 children with left USCP. Children were classified following the Manual Ability Classification System [34] as levels I (*n* = 16), II (*n* = 50), or III (*n* = 9). Brain lesions as observed on available MRI (*n* = 69) were subcortical and cortical lesions of frontal/parietal/temporal areas (n = 4), subcortical and cortical lesions of frontal/parietal/temporal areas and insula (*n* = 13), subcortical and cortical lesions of frontal/parietal/temporal/occipital areas and insula (*n* = 9), subcortical and cortical lesions of parietal/occipital/temporal areas (*n* = 3), subcortical and cortical lesions of parietal/temporal areas (*n* = 9), and subcortical without cortical lesions (*n* = 31). Ophthalmological examinations were available in a subsample of 13 children. Three children had a visual field defect as measured using Goldmann visual field perimetry (2 hemianopsia, 1 quadranopsia; see Table 2), and 3 children had a deficit of eye motility [37]. Two of those three children had at least 1 abnormal result upon testing of visuospatial attention. Due to the small size of the subsample, no further statistical analyses were performed. Six children were taking antiepileptic drugs and had no clinically observable seizures in their recent medical history or at the time of testing.

The individual clinical data and individual results of the visuospatial attention assessments are shown in Table 2.

### 4.2. Prevalence of Visuospatial Attention Deficits in Children with USCP

Sixty percent of the children presented with abnormal values of at least one visuospatial attention test. 28% of the children with USCP presented with abnormal values of two or more visuospatial attention tests. 10.7% of the children presented with abnormal values of three or more visuospatial attention tests. No association was found between age groups and the presence of abnormal visuospatial assessments (chi-square, all *p* > 0.390). No significant association was observed between the MACS level and the presence of abnormal visuospatial attention assessments (chi-square, all *p* > 0.054). No significant association was observed between the GMFCS level and the presence of abnormal visuospatial attention assessments (chi-square, all *p* > 0.402).

Differences in the percentage of children with deficits were observed depending on the timing of the lesion: compared to children with periventricular lesions, a larger percentage of children with corticosubcortical lesions presented with visuospatial attention deficits (*χ*^2^(3, *n* = 32) = 16.655; *p* = 0.001). The prevalence of abnormal visuospatial attention assessments was not different between the different brain lesion localizations (chi-square, all *p* > 0.612).

No differences were observed for the prevalence of abnormal visuospatial attention assessments between children taking antiepileptic medication and those without (chi-square, all *p* > 0.663).

A significant association was observed between the prevalence of abnormal star cancellation and the prevalence of abnormal Ogden figure copy (*χ*^2^(1, *n* = 14) = 11.193; *p* = 0.010). No other significant associations between abnormal visuospatial attention assessments were found (all *p* > 1.000).

**Figure 1:**
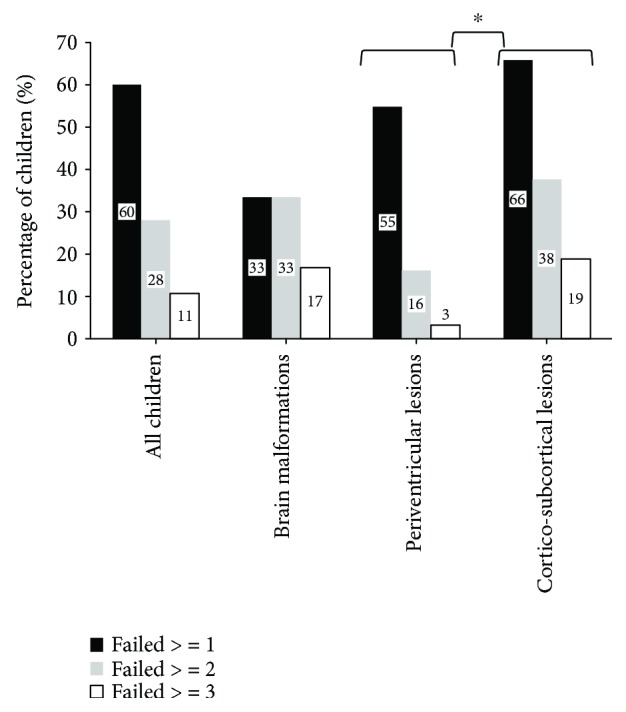
Percentage of children presenting with a visuospatial attention deficit in the whole sample.

**Figure 2:**
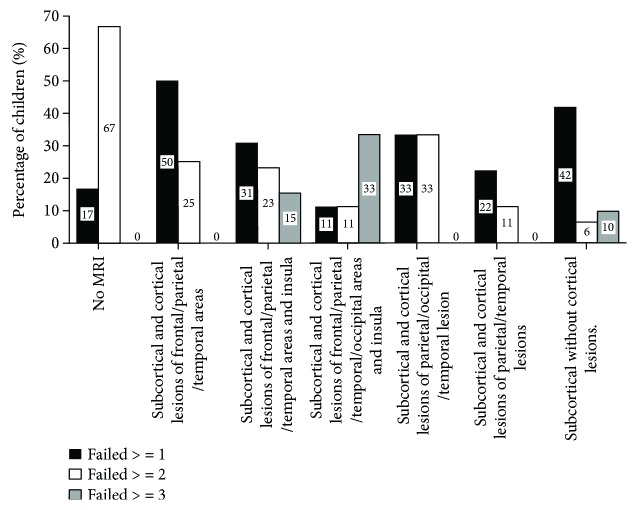
Percentage of children presenting with a visuospatial attention deficit in function of lesion localization.

Figure 1 shows the prevalence of abnormal values for one, two, three, or more visuospatial attention tests in the whole sample of children with USCP as well as in children classified by lesion timing.  Figure 2 shows the prevalence of abnormal visuospatial attention assessments classified by lesion localization.

### 4.3. Prevalence of Abnormal Findings in Each of the Visuospatial Attention Tests in Children with USCP

**Figure 3:**
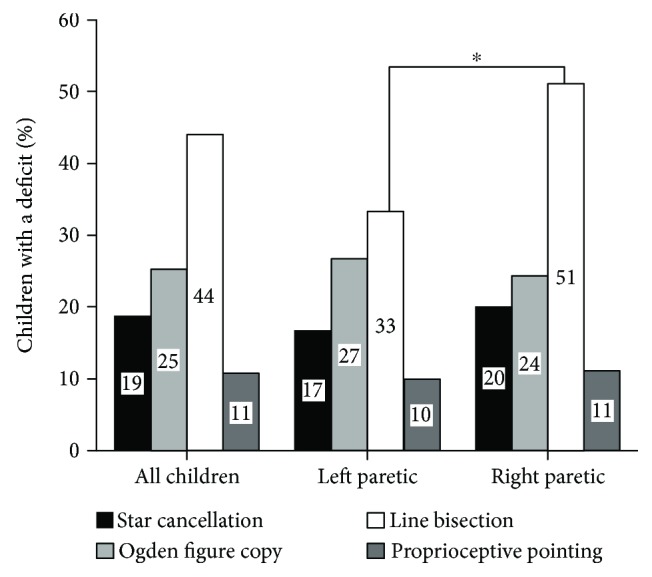
Percentage of children with USCP with abnormal values in each visuospatial attention test for the whole sample and for children with left or right USCP. Chi-square ^∗^*p* < 0.050.

**Figure 4:**
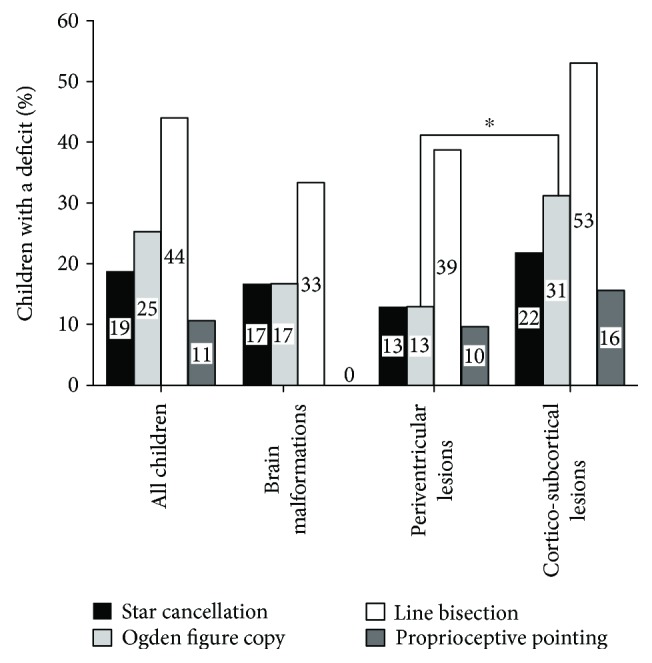
Percentage of children with USCP with abnormal values in each visuospatial attention test for the whole sample and for children with brain malformation, periventricular lesion, or corticosubcortical lesions. Chi-square ^∗^*p* < 0.050.

The prevalence of abnormal values in each of the four visuospatial attention tests in children with USCP is described in Figures 3 and 4.

#### 4.3.1. Star Cancellation

**Figure 5:**
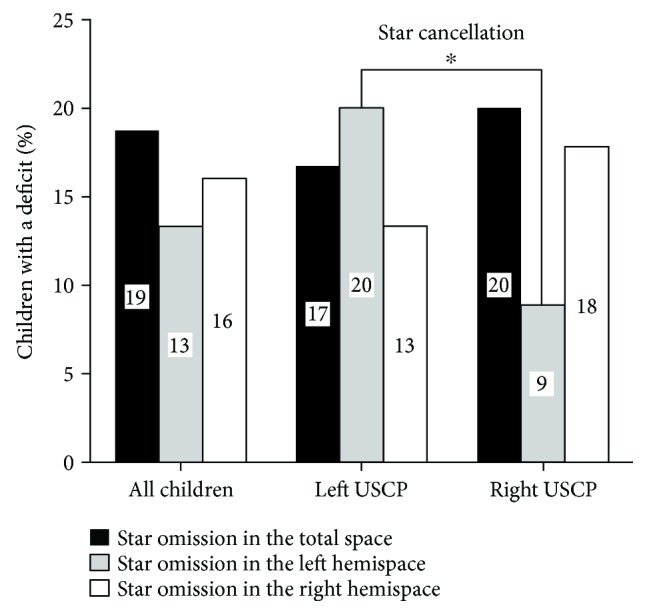
Percentage of children with USCP with abnormal findings in the star cancellation test for each hemispace (star omission in the total space, star omission in the left hemispace, and star omission in the right hemispace). Chi-square ^∗^*p* < 0.050.

18.7% of the children (number of tested children = 75) presented with abnormal values. The absolute difference between left and right omitted stars was significantly different from “zero,” indicating that children omitted more stars on one side than on the other (children with left USCP: *t*(1, 29) = 2.769; *p* = 0.01; children with right USCP: *t*(1, 44) = 4.100; *p* < 0.0001) (Figure 4). When the prevalence of abnormal values was compared between children with left and right USCP, children with a left USCP presented significantly more abnormal values for left omitted stars than children with right USCP (*χ*^2^(1, *n* = 30) = 4.559; *p* = 0.033) (Figure 5). The prevalence of abnormal values was not significantly different between children with periventricular or corticosubcortical lesion for the total number of omitted stars: *χ*^2^(1, *n* = 32) = 2.294; *p* = 0.130. In the number of right omitted stars, the prevalence of abnormal values was significantly larger in children with corticosubcortical lesions than in children with periventricular lesions (*χ*^2^(1, *n* = 32) = 49.095; *p* < 0.001). The prevalence of abnormal values did not differ by lesion location (all *p* > 0.300).

#### 4.3.2. Ogden Figure Copy

25.3% of the children (number of tested children = 75) presented with abnormal values. The prevalence of abnormal values was not significantly different between children with right and left USCP: *χ*^2^(1, *n* = 30) = 0.084; *p* = 0.773. The prevalence of abnormal values was significantly higher in children with corticosubcortical lesions than in children with periventricular lesions (*χ*^2^(1, *n* = 32) = 9.590; *p* = 0.002) (Figure 4). The prevalence of abnormal values did not differ by lesion location (all *p* > 0.600).

#### 4.3.3. Line Bisection

44% of children (number of tested children = 75) presented with abnormal values. Twenty-five children were above the upper bound of the reference range (i.e., bisection deviated towards the nonparetic hemispace), and 8 children were below the lower bound of the reference range (i.e., bisection deviated towards the paretic hemispace). Children with right USCP had abnormal values more often than children with left USCP (*χ*^2^(1, *n* = 45) = 6.427; *p* = 0.011; children with right USCP = 51.1% and children with left USCP = 33.3%). In the line bisection test, the prevalence of abnormal values was not significantly different in function of lesion timing (*χ*^2^(1, *n* = 32) = 2.807; *p* = 0.094). The prevalence of abnormal values did not differ by lesion location (all *p* > 1.00).

#### 4.3.4. Proprioceptive Pointing

10.6% of children (number of tested children = 75) presented with abnormal values: 7 children deviated towards the nonparetic hemispace, and 1 child deviated towards the paretic hemispace. The prevalence of abnormal values was not significantly different between children with right and left USCP: *χ*^2^(1, *n* = 30) = 0.037; *p* = 0.848. The prevalence of abnormal values was not significantly different between children with predominant white matter lesions and predominant grey matter lesions: *χ*^2^(1, *n* = 32) = 1283; *p* = 0.257. The prevalence of abnormal values did not differ by lesion location (all *p* > 1.00).

## 5. Discussion

The aim of this study was to investigate the prevalence of visuospatial attention deficits among children with USCP using both ego- and allocentric tests, taking into consideration the affected hemibody. A majority of children with USCP presented with abnormal visuospatial attention as 60% of our sample scored outside the reference values for at least one visuospatial attention test. In addition, the results indicated a difference between children with left and right USCP. Children with a left USCP showed predominantly an egocentric impairment and children with a right USCP showed mainly an allocentric deficit. Lesion timing also had an influence on the prevalence of visuospatial attention deficits: children with corticosubcortical lesions presented more frequent visuospatial attention deficits than children with periventricular brain lesion. A significant association was observed between an abnormal star cancellation test and an abnormal Ogden figure copy in children with USCP, as previously reported in typically developing children [18, 44].

The originality of the present study lies within the large school-aged population of exclusively children with USCP, investigating both ego- and allocentric visuospatial attention. Previous studies assessing visuospatial attention abilities included children with all types of acquired brain lesions and used a limited number of tasks: the Teddy Bear cancellation task [21] or a spatial exploration task [20] or the clock drawing test [25]. The present study confirms previous findings in egocentric visuospatial attention assessments [20–23], while giving a more complete overview of visuospatial attention deficits in children with USCP.

More than half of the children participating in this study presented with abnormal values for at least one visuospatial attention test and almost one-third of the sample for two or more tests. The presence of visuospatial attention deficits and in particular neglect of one side of space could be relevant for the rehabilitation process in children with USCP. Evidence shows that visuospatial attention interacts with motor function, for instance, during eye-limb coordination [10]. In this way, an early motor deficit could have an impact on the development of the attentional system [45], for example, children with spastic diplegia have shown impairments in visual orientation tasks [46]. Ideally, a global deficit of attention or executive functioning should have been ruled out by a control task. This was not possible in the present study because children were included as participants of an intensive rehabilitation program and were subjected to a large number of assessments during the limited amount of time available before the start of rehabilitation program. However, a global attentional problem appears as improbable for two reasons: (1) visuospatial attention deficits were lateralized and (2) very few children showed abnormal results in all of the four visuospatial attention assessments.

As with other executive functions, visuospatial attention may develop with age. In typically developing children, the performance on some visuospatial attention tasks was shown to mature with age [18]. Previous studies have shown that younger children with CP may present with more visuospatial neglect than older children [23, 25]. The absence of any effect of age on the prevalence of visuospatial attention deficits in the present findings does not preclude that age still may influence their visuospatial abilities in children with CP. The present study was not designed to examine an effect of age: the age range of the included children was too narrow and the visuospatial attention assessments were administered only once in each subject. Hence, no age-related differences were observed.

Visuospatial attention deficits were more frequently observed in children with corticosubcortical lesions than in children with periventricular lesions. Previous studies suggested that children with cortico/subcortical lesion generally present with larger lesions than children with periventricular lesions. Also in these children, more associations are observed between lesion characteristics and clinical outcomes [6, 47]. Mailleux et al. [6] reported frequent and stronger associations between lesion characteristics (size, localization, and extent) and motor function in children with cortico/subcortical lesion than in children with periventricular lesions. Impaired upper extremity function [47] is also more common in CP children with cortical/subcortical lesion compared to periventricular lesions. This overall larger prevalence of deficits in children with cortico/subcortical lesions could be explained by the timing of the lesion. Cortico/subcortical lesions typically arise at the end of the 3^rd^ trimester of gestation [36]: the later the lesion, the less likely it may allow for efficient reorganization/rewiring of affected functions in the brain.

Visuospatial attention deficits were observed in children with right as well as with left brain lesions. This clinical picture is very different from the one in adults demonstrating mainly hemineglect with right brain lesions [48] due to the lateralization of visuospatial abilities within the right hemisphere [49]. Similar results in children either with left or right brain lesions have been reported previously by Thareja et al. [23]. The fact that left brain lesions can lead to an alteration of visuospatial abilities in children with USCP can be explained by the important cerebral reorganization occurring after an early brain lesion. This observation may be explained by the “crowding hypothesis” [24, 50]: a left hemispheric lesion can shift the areas related with language from the left to the right hemisphere, thus affecting visuospatial function. Lidzba et al. [24] highlighted a correlation between the reorganization of language function in the right hemisphere and visuospatial performance in children with early cerebral lesions.

Differences in the type of hemineglect were observed between children with left and right USCP. In the star cancellation test (assessing mainly egocentric neglect), children with left USCP omitted more stars on the left side than on the right side and were more often outside the normative values for the number of left omitted stars than children with right USCP. On the other hand, children with right USCP more frequently presented with abnormal values of the line bisection test compared to children with left USCP, suggesting more often allocentric visuospatial impairment [19]. It has been suggested that different brain substrates are linked to egocentric and allocentric neglect: egocentric neglect being linked to the fronto-parieto-temporal network, while allocentric neglect being related to the parieto-temporo-occipital network [51]. Specifically, egocentric representation has been related with activation in the medial part of the left superior parietal lobe and the allocentric representation with activation in the right parietal lobe, occipitotemporal cortex, and hippocampal regions [52]. Besides the side of hemispheric lesion, specific characteristics of the brain lesion and postlesional brain reorganization and development also may explain the differential visuospatial attentional impairments: larger brain lesions have been observed in children with right USCP than in children with left USCP [53, 54]. However, in the present study, the presence of abnormal visuospatial assessments was not related to the localization of brain lesions. It must be noted that brain MRIs were not acquired for this study specifically and lesion localizations were interpreted post hoc from available MRIs. More relevant imaging data including fMRI probably could clarify how lesion characteristics and brain reorganization and maturation relate to the development of visuospatial abilities in children with CP.

Among the limitations of the study, it is important to acknowledge the partial availability of ophthalmological examinations in the study sample. Previous studies have investigated the development of visual abilities through childhood as well as the importance of measuring such abilities in children with CP [55, 56]. It is not possible to establish the relationship between visual impairment and visuospatial attention deficits with only 20% of the study sample having received an ophthalmological examination during clinical follow-up. Furthermore, visual field testing is rarely performed in a clinical setting and even more seldom in young children. We can only speculate on how potential visual field defects may have influenced visuospatial attention in our study sample. Adults with hemianopsia performing the line bisection test show an inverse pattern compared to adults with hemineglect [57–61]. Indeed, hemineglect patients bisect towards the ipsilesional side of the lines, whereas hemianoptic patients bisect towards the contralesional side of the lines [57, 58]. Deficits in oculomotor function may also impair visuospatial attention. Correct saccadic movements have been described as important for the development of visuospatial attention [10]. Ego et al. [62] have described a relatively preserved oculomotor function in children with CP which evolves with age to reach almost the same performance as typically developing children. It appears as less probable that deficits in oculomotor function would impair the possibility to scan the environment and lead to deficits of visuospatial attention. Future studies should include a complete ophthalmological examination for all study subjects to exclude underlying impairments of vision as a substrate for visuospatial attention deficits.

In addition, although children of this study attended school in the same grade as their typically developing peers of the same age, intellectual quotient (IQ) per se was not tested and thus we cannot exclude an influence of IQ on our results. This is especially a concern since an interplay between cognitive function and visuospatial abilities has been previously described [10, 27, 63].

This study gives a better insight in the prevalence of visuospatial attention deficits in children with USCP and highlights that visuospatial deficits are common among children with USCP and more frequent in children with cortico/subcortical lesions than in children with periventricular lesions. In order to properly diagnose these deficits, both egocentric and allocentric visuospatial attention tests are needed. Children with right and left USCP do not present the same type of visuospatial attention deficits: left USCP is more linked to egocentric neglect while right USCP is more linked to allocentric neglect. Also, visuospatial attention deficits observed in children with CP were different from those reported in adult patients. This may be due to the nature of brain lesions as well as the process of dynamic brain (re)organization [24, 32]. Though the present results did not indicate any relationship between age and visuospatial abilities in a cross-sectional sample of children with CP, future studies should further investigate the evolution of visuospatial attention deficits in the function of lesion characteristics and brain development in children with cerebral palsy. Indeed, it is possible that spatial deficits observed at a young age may become clinically insignificant at a later stage [18, 23, 25]. Future research should thus include medical imaging in combination with visuospatial and other neuropsychological assessments in a longitudinal perspective. The present findings may help in improving the rehabilitation of children with USCP as visuospatial abilities are critical for motor skill learning and motor control. Depending on the side of the brain lesion, children may show differential responses related to the lateralization aspect of these deficits. Different rehabilitation interventions have been described in adult patients such as vestibular stimulation or prismatic rehabilitation [64–66]. Prismatic rehabilitation has been reported as feasible in children with USCP [43]. Future studies should therefore investigate the effectiveness of prismatic rehabilitation applied to children with USCP for improving visuospatial neglect and possibly motor skill learning.

## Figures and Tables

**Table 1:** Demographic and clinical characteristics of children with unilateral spastic cerebral palsy.

	More affected upper extremity		
	Left	Right	All
Age	9 y 5 m (3 y)	9 y 1 m (2 y 11 m)	9 y 3 m (2 y 11 m)
Gender (*n*)			
Female	9	24	33
Male	21	21	42
Lesion timing (*n*)			
Brain malformation	4	2	6
Periventricular white matter lesion	14	17	31
Cortical/subcortical lesion	10	22	32
NA	2	4	6
MACS (level)			
Level I	7	9	16
Level II	22	28	50
Level III	1	8	9
GMFCS (level)			
Level I	27	37	64
Level II	3	8	11
Total (*n*)	30	45	75

MACS: Manual Ability Classification System; GMFCS: Global Motor Function Classification System.

**Table 2:** 

Subject	Age	Total omitted stars (*n*)	Ogden figure copy (score)	Line bisection: average error (%)	Proprioceptive pointing: average error (%)	MACS (level)	GMFCS (level)	Lesion timing	Lesion side	Lesion localization	Antiepileptic drug	Ophthalmologic deficits
1	5	4	1	20.8	−3.0	1	1	PWM/PVL	Left	c	NA	NA
2	5	16	4	−8.7	3.3	2	1	GMI	Right	e	NA	NA
3	6	3	0	−18.5	−4.3	2	1	GMI	Right	a	NA	NA
4	6	27	4	−0.1	2.5	1	1	No MRI	Right	a	NA	NA
5	6	0	0	−0.5	3.0	1	2	GMI	Left	c	NA	NA
6	6	12	4	−1.5	1.0	2	2	GMI	Left	c	z	O
7	6	9	3	38.5	4.0	2	1	GMI	Left	d	NA	NA
8	6	1	0	6.2	0.8	1	1	PWM/PVL	Left	f	NA	NA
9	6	8	2	−3.2	1.8	2	1	GMI	Left	f	NA	NA
10	6	0	0	−1.5	0.3	2	2	PWM/PVL	Left	g	NA	O
11	6	4	1	16.0	−1.0	3	1	PWM/PVL	Left	g	NA	NA
12	6	41	4	32.9	1.7	2	1	GMI	Left	g	NA	NA
13	6	3	2	19.0	7.5	1	1	PWM/PVL	Left	g	NA	NA
14	6	3	1	3.0	27.0	2	1	PWM/PVL	Right	g	NA	NA
15	6	1	1	11.8	6.0	1	1	PWM/PVL	Right	g	NA	NA
16	7	2	1	7.9	0.3	2	1	GMI	Left	b	NA	O
17	7	1	0	6.5	−5.8	2	1	GMI	Left	b	NA	NA
18	7	5	1	19.4	−5.5	2	1	GMI	Left	c	NA	NA
19	7	1	0	9.5	2.5	2	1	PWM/PVL	Left	c	NA	NA
20	7	8	2	14.8	6.8	2	2	GMI	Left	d	NA	H/St
21	7	1	1	3.2	0.5	2	1	GMI	Left	d	NA	NA
22	7	6	1	12.0	2.5	2	1	GMI	Left	d	NA	NA
23	7	5	2	8.5	1.3	2	1	GMI	Left	f	NA	NA
24	7	2	0	−10,8	−0,5	2	1	MCD	Right	f	z	NA
25	7	0	0	−4.2	5.3	1	1	MCD	Right	f	NA	NA
26	7	5	2	13.3	1.0	2	1	PWM/PVL	Left	g	NA	Exo/M-1
27	7	6	2	4.5	−0.3	2	1	No MRI	Left	g	NA	NA
28	7	2	1	−10.1	−4.3	2	2	GMI	Right	g	NA	Q/Exo-R Hyper
29	7	9	0	−7.2	4.0	1	1	PWM/PVL	Right	g	NA	O
30	7	4	1	−8.7	6.7	3	1	PWM/PVL	Right	g	NA	NA
31	7	25	4	−30.6	0.0	2	1	MCD	Right	g	NA	NA
32	7	6	0	−3.9	−0.8	2	1	PWM/PVL	Right	g	NA	NA
33	8	0	1	−4.8	−3.5	2	1	GMI	Right	b	NA	St/Eso
34	8	14	2	−17.8	−0.7	2	1	PWM/PVL	Right	c	NA	NA
35	8	0	0	−7.1	−0.5	2	1	GMI	Right	c	NA	NA
36	8	0	0	−2.7	5.0	3	1	GMI	Left	d	NA	NA
37	8	4	1	−6.3	6.3	2	1	GMI	Right	f	NA	NA
38	8	0	0	13.8	−1.0	2	2	GMI	Left	g	NA	NA
39	8	0	0	3.7	0.3	1	1	PWM/PVL	Left	g	NA	NA
40	8	0	0	4.5	1.8	1	1	PWM/PVL	Left	g	NA	NA
41	8	9	1	−9.1	−15.0	1	1	PWM/PVL	Right	g	NA	NA
42	8	31	2	−5.4	11.3	2	1	GMI	Right	g	NA	NA
43	9	7	0	8.0	4.3	3	1	GMI	Left	c	NA	NA
44	9	1	0	9.5	1.0	3	1	GMI	Left	c	NA	NA
45	9	0	1	−5.1	0.5	3	1	No MRI	Left	c	NA	NA
46	9	2	2	7.5	4.0	3	1	GMI	Left	d	NA	NA
47	9	4	0	−0.4	−0.4	2	2	MCD	Left	e	z	O
48	9	0	0	1.2	1.3	2	1	PWM/PVL	Left	g	NA	NA
49	9	5	0	11.5	−0.8	2	1	PWM/PVL	Left	g	NA	NA
50	10	2	2	28.0	4.8	2	2	GMI	Left	d	y	NA
51	10	0	1	−0.6	−1.8	2	2	GMI	Right	e	y	NA
52	10	0	0	−0.9	−0.8	2	2	PWM/PVL	Left	g	NA	O
53	10	2	0	−1.3	6.0	2	1	PWM/PVL	Left	g	NA	NA
54	10	2	0	8.2	1.3	1	1	PWM/PVL	Left	g	NA	NA
55	11	1	0	−2.6	−0.5	2	1	No MRI	Left	a	NA	NA
56	11	3	0	6.4	1.8	1	1	GMI	Left	b	NA	NA
57	11	0	0	−2.8	0.0	1	1	GMI	Right	c	NA	O
58	11	1	0	−8.5	−4.0	2	1	GMI	Right	d	NA	NA
59	11	0	0	−1.0	2.3	2	2	PWM/PVL	Right	g	NA	NA
60	11	1	0	−5.2	−1.8	2	1	PWM/PVL	Right	g	NA	St/L-Hyper/M1/M-2
61	11	1	0	−11.4	1.8	2	1	PWM/PVL	Right	g	NA	NA
62	12	13	2	−0.6	−1.5	2	1	PWM/PVL	Left	a	NA	NA
63	12	1	2	−7.6	−1.8	2	1	No MRI	Right	a	NA	NA
64	12	0	0	−0.1	1.3	2	1	MCD	Left	f	NA	NA
65	12	0	0	2.3	−3.7	2	1	GMI	Left	f	NA	NA
66	12	4	0	2.0	−4.0	1	1	PWM/PVL	Left	g	NA	NA
67	12	0	0	−6.8	−1.7	1	1	MCD	Right	g	NA	NA
68	13	0	1	15.8	9.3	3	1	GMI	Left	c	NA	NA
69	13	1	0	6.3	0.0	2	1	PWM/PVL	Right	g	NA	NA
70	14	0	0	−2.6	0.0	2	1	PWM/PVL	Right	g	NA	NA
71	15	1	0	7.6	0.0	2	1	No MRI	Left	a	y; x	NA
72	15	0	0	3.2	1.5	2	1	PWM/PVL	Left	g	NA	NA
73	16	0	0	−6.8	−3.5	2	1	PWM/PVL	Right	d	NA	H/St/ESO/M-3
74	17	0	1	−12.8	1.0	2	1	PWM/PVL	Right	c	NA	NA
75	17	0	0	0.8	−4.7	3	1	GMI	Left	f	NA	NA

Lesion timing: MCD: brain malformation; PWM/PVL: periventricular white matter lesion; GMI: cortical/subcortical lesion. Lesion localization: a: no MRI; b: subcortical and cortical lesions of frontal/parietal/temporal areas; c: subcortical and cortical lesions of frontal/parietal/temporal areas and insula; d: subcortical and cortical lesions of frontal/parietal/temporal/occipital areas and insula; e: subcortical and cortical lesions of parietal/occipital/temporal lesion; f: subcortical and cortical lesions of parietal/temporal lesions; g: subcortical without cortical lesions. Antiepilectic drug: z: valproate; y: carbamazepine; x: lamotrigine. Ophthalmologic deficit: NA: no available data; O: no deficit; H: hemianopsia; Q: quadranopsia; St: stereoscopic vision deficit; Exo: exotropia; Eso: esotropia; R Hyper: right hypertropia; L Hyper: left hypertropia; M-1: eye motility deficit in the left eye; M-2: eye motility deficit in the right eye; M-3: presence of gaze-evoked nystagmus.

## Data Availability

The data used to support the findings of this study are available from the corresponding author upon request.

## Additional Points

*What this paper adds*. (i) Insight on the prevalence of visuospatial deficits among children with USCP. (ii) Highlights differences in visuospatial abilities between children with left and right USCP and their relationship with allocentric and egocentric visuospatial representations.
